# Bubble Growth in Poly(methyl methacrylate) and Carbon Dioxide Mixture

**DOI:** 10.3390/polym11040648

**Published:** 2019-04-09

**Authors:** Jie Chu, Xiaofei Xu

**Affiliations:** Center for Soft Condensed Matter Physics and Interdisciplinary Research, Soochow University, Suzhou 215006, China; jchu@stu.suda.edu.cn

**Keywords:** poly(methyl methacrylate), carbon dioxide, mixture, bubble nucleation, molecular dynamics simulation, free volume

## Abstract

In this paper, we study bubble nucleation and growth in a poly(methyl methacrylate) and CO${}_{2}$ mixture by molecular dynamics simulations. It is known in the foaming industry that the bubble size has a more uniform distribution with a higher start-up pressure. The real physical reason remains unclear. In this work, we found that the free volume-rich polymer segments could adsorb many small-size bubbles in the region close to the polymer chain. The existence of these small bubbles limits the number of free CO${}_{2}$ molecules, which is helpful for bubble stabilization. Moreover, the free volume of polymer segments decreases with an increase of the start-up pressure. As a result, the size of the large bubbles becomes more uniform with a higher startup pressure.

## 1. Introduction

The importance of polymeric foam in our society can be understood by the steady growth in consumption of polymer materials in the last 50 years [1]. Polymer foaming processes have a wide application in chemical engineering and materials science [2,3,4,5]. A typical polymeric foaming process consists of dissolving a gas phase into a polymer melt, then foaming the gas (i.e., bubble nucleation) and, subsequently, solidifying the mixture [5]. Although there have been many studies on the foaming process [6,7,8,9,10,11], few of them focus on the post-nucleation stage of the solidification process. Available publications have used a mesocopic approach to study bubble growth on a micro-scale [12,13,14,15]. In this work, we will explore this problem on the nano-scale, by using molecular dynamics simulations.

To prevent bubbles condensing or collapsing back into the liquid phase, foams need to be solidified after bubble nucleation [5,7]. The solidification process starts after exposing foams to their surroundings. As the system is cooling down, the melt viscosity increases to slow down bubble movement. As a result, the bubbles have a much longer lifespan, giving a permanent and definite porous structure. This process can take anywhere from seconds to days until a thermal and kinetic equilibrium between the foam and the surroundings is reached. The thermodynamic, kinetic, and transport properties of the gas and melt viscosity are of vital importance in solidification [8,9].

There are several unsolved issues in the solidification process. The bubbles keep growing until the cell surface is solidified. When expansion increases, contact between neighboring bubbles occurs. The shared wall becomes thinner, and the cell may rupture into an open cell [3]. Manufacturing foams with fewer open cells is a challenge in foaming industry [5]. In addition, gas molecules close to the boundary or surface can escape from the surface. Surface escape effects will decrease the foaming efficiency [16]. It is important to explore these issues by using molecular simulation, which is useful in controlling the cell size and shape of foams by altering the physical conditions (such as *T*, *P*, or the rate of dropping pressure) in industry.

As an environmentally-benign blowing agent, carbon dioxide (CO${}_{2}$) has widespread application in foaming processes. In this work, we study bubble growth and coalescence in a mixture of poly(methyl methacrylate) (PMMA) and CO${}_{2}$ by molecular dynamics simulations. We focus on the post-nucleation stage of the solidification process. It is known, in the foaming industry, that the bubble size has a more uniform distribution with a high start-up pressure [5]. We will explore the physical reasons for this phenomenon by collecting data for the bubble dynamics.

## 2. Simulation Methods

**Figure 1:**
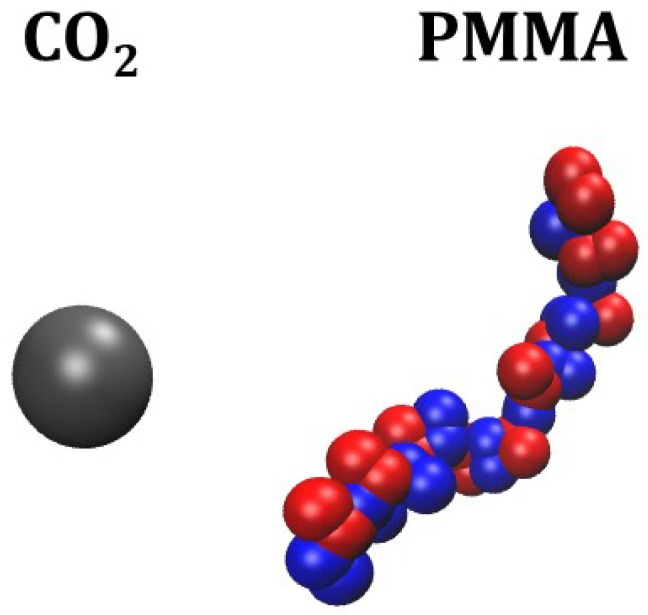
Molecular model. There are two types of coarse-grained bead in the poly(methyl methacrylate) (PMMA) chain. The main chain atoms form bead A (red color) and the ester side-group forms bead B (blue color).

We consider a mixture of PMMA and CO${}_{2}$ in a cubic simulation box with periodic boundary in three dimensions; CO${}_{2}$ is coarse-grained by a spherical bead, (see Figure 1). In order to obtain a good CO${}_{2}$ model, we perform simulation in a NVT ensemble of pure CO${}_{2}$ molecules. The simulation box is cubic with dimensions 30σ×30σ×30σ, where σ is the diameter of CO${}_{2}$.

**Figure 2:**
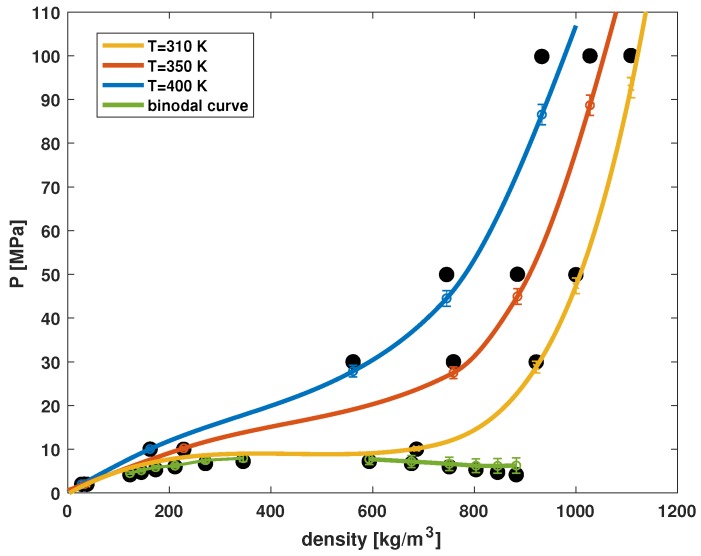
Comparison between simulation predictions (solid lines) and experimental data (solid points). The green line is the liquid–vapor co-existence curve, and the other solid lines are the isotherms.

The number of CO${}_{2}$ molecules in the simulation box is determined by fixing the experimental density at a given temperature:

(1) $$N=\frac{\rho_{m}N_{A}L^{3}}{M},$$

where $\rho_{m}$ is the mass density, *M* is the molar mass, *L* is the box length, and $N_{A}$ is Avogadro’s constant. We, then, run the simulation by molecular dynamics in LAMMPS using a velocity-Verlet algorithm with time step Δt=0.01τ, where τ=0.55 ps is the time scale. The system pressure is evaluated after reaching dynamic equilibrium. The molecular parameters of CO${}_{2}$ are obtained by fitting the simulation results with experimental data [17,18]. In Figure 2, we show two types of data: The isotherms and the liquid–vapor co-existence curve. For the liquid–vapor co-existence curve, the right branch is the liquid phase and the left branch is the vapor phase. At a given pressure, the liquid phase co-exists with the vapor phase. Our model well predicts the phase behavior of CO${}_{2}$.

PMMA is modeled by a flexible chain with two types of beads (see Figure 1). The main chain atoms in PMMA are bead A and the ester side-group is bead B. The parameters (diameter σ, energy strength ϵ, bond length, torsion, and bending angle) of beads A and B are obtained by fitting the force field with a fully-explicit atom model [19,20]. We list ϵ and σ values for all beads in Table 1. The data for bond length, bending, and torsional angle can be found in [19].

There are 1000 CO${}_{2}$ molecules and 50 PMMA chains of length N=40 in the simulation box. The simulation box is cubic with dimensions 100σ×100σ×100σ. The intermolecular potentials (between pairs of beads) are described by a Lennard-Jones potential

(2) $$u_{ij}\left(r\right)=4\epsilon_{ij}\left[{\left(\frac{\sigma_{ij}}{r}\right)}^{12}-{\left(\frac{\sigma_{ij}}{r}\right)}^{6}\right],$$

where $\epsilon_{ij}=\sqrt{\epsilon_{i}\epsilon_{j}}$ and $\sigma_{ij}=\left(\sigma_{i}+\sigma_{j}\right)/2$. We perform the simulation of the mixture in a NPT ensemble at T=310 K. We run the simulation with a time step of Δt=2fs and a cutoff distance for the potential at $r_{c}=5\sigma$.

**Figure 3:**
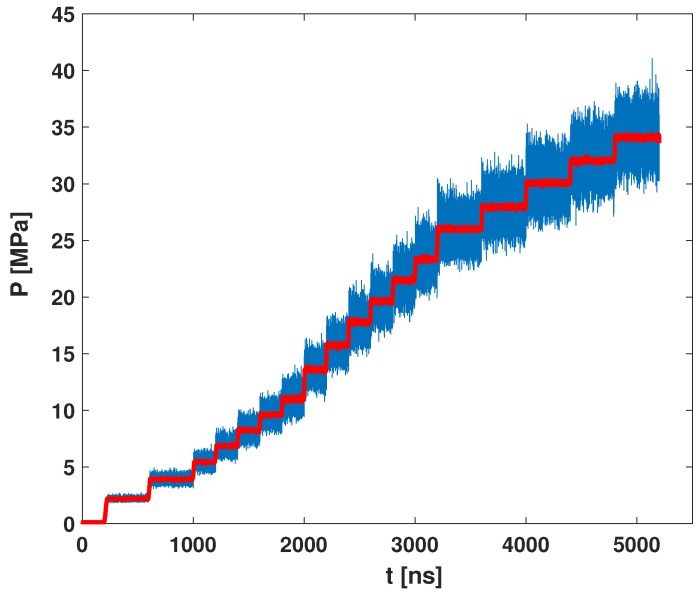
System pressure in the simulation. We increase the pressure step-by-step to reach the startup pressure for nucleation. The blue line is the raw data and the red line is the ensemble average value over time.

Nucleation is the initial stage of phase transition from a metastable state to a stable state. In order to prepare the initial metastable state, we increase the system pressure to a high value (30 MPa or more), and quickly drop the pressure to 0.1 MPa. The system will be metastable and then starts to nucleate. However, the molecular configuration is hard to equilibrate if we directly increase the pressure to those high pressures, because molecules at high pressure are hard to move in the simulation box. Rather than increasing directly, we gradually increase system pressure, step by step, from ambient pressure to get a good configuration for nucleation (see Figure 3). At each pressure step, we run the simulation at least $2\times 10^{8}$ steps to reach a dynamic equilibrium, which gives a reasonable configuration. The simulation at next pressure step starts from this configuration. Finally, we reach a high pressure (of 30 MPa or more). The configuration obtained in this way works well for nucleation in our simulation.

## 3. Results and Discussion

**Figure 4:**
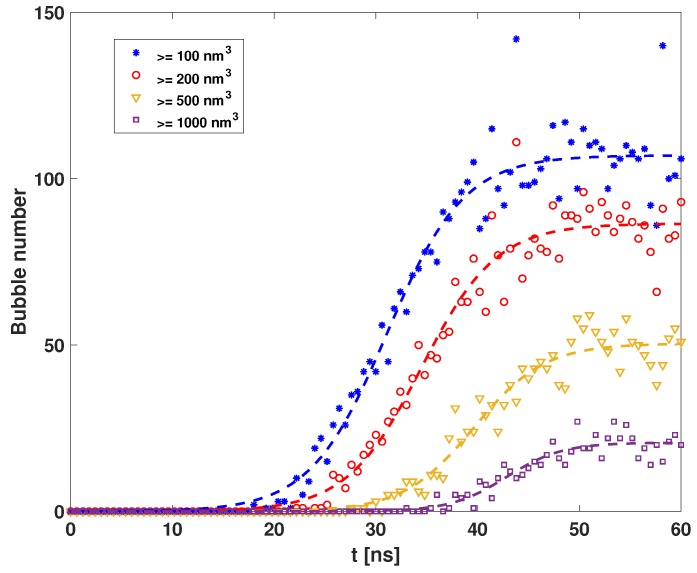
Time evolution of bubble number with a start-up pressure of 30 MPa. The dashed line is the fitting curve. At t=0 ns, the system starts nucleation.

The data below are statistics for CO${}_{2}$. We did not use any empirical model to collect data. The bubble has a free shape in the polymer foam. For simplicity, in this study we define one CO${}_{2}$ bubble to be the biggest spherical cavity consisting only of CO${}_{2}$ molecules. In Figure 4, we show the time evolution of the bubble number in the simulation box, up to various sizes. Distinct bubbles are identified so that they did not overlap. As shown, there were three stages in our simulation: pre-nucleation, nucleation, and post-nucleation stages. After the pressure drop (t=0 ns), due to the free energy difference, the metastable bulk phase started to nucleate to a stable phase. The CO${}_{2}$ molecules assembled to form a nucleus. This was the pre-nucleation stage (t<20 ns). With the nucleus growing, the bubble had to overcome the nucleation barrier to form a larger bubble. It is known that nucleation is an activated process, involving free energy barriers between two states. For fluctuations with energies lower than the barrier, the nucleus shrunk. Only when the fluctuations were sufficiently large—larger than that required to reach the critical nucleus—did the nucleus grow spontaneously [6]. This was the nucleation stage (20<t<40 ns). In the post-nucleation stage, the bubbles fused to grow into a larger bubble. Finally, the bubble stopped growing when the pressure and temperature inside were in equilibrium with the surrounding environment. The plateau seen for t>40 ns in these curves corresponds to the post-nucleation process.

**Figure 5:**
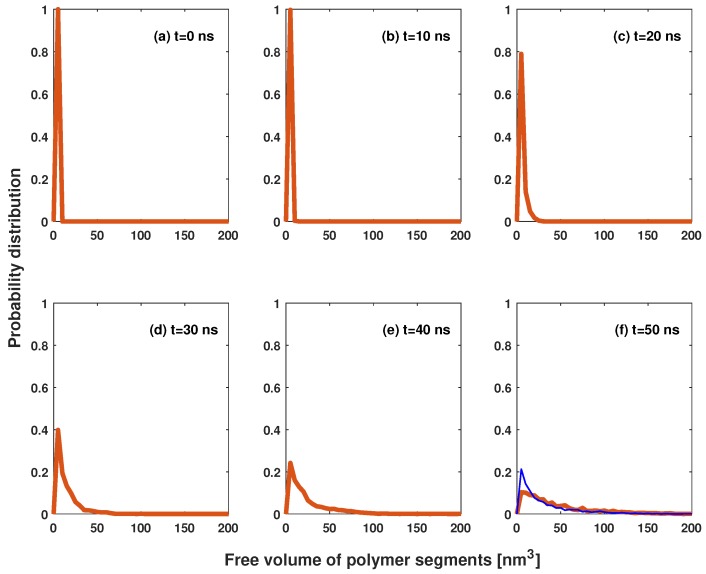
Free volume distribution for polymer segments. The red solid lines are the data at a start-up pressure of 30 MPa; and the blue thin line in panel (f) is the data at 34 MPa start-up pressure.

CO${}_{2}$ molecules move freely in cells made of surrounding molecules. The volume within which the center of one molecule moves is defined as the free volume [21]. Free volume plays an important role in polymeric foaming. Free volume-rich polymers mix more favorably with CO${}_{2}$ [22]. A high fraction of free volume decreases melt viscosity [23], which is not good for bubble stability in the post-nucleation stage; it becomes easy for the CO${}_{2}$ molecules to escape from the bubbles. Figure 5 shows the probability distribution [24] of the free volume for polymer segments at various times. In the pre-nucleation/nucleation stages, most polymer segments have a very small free volume, due to the high pressure of the system. Free volume increases with bubble growth. In the post-nucleation stage, some segments have a large free volume. Our simulations show that the free volume of polymers decreases with a higher start-up pressure; see the blue line in Figure 5f.

**Figure 6:**
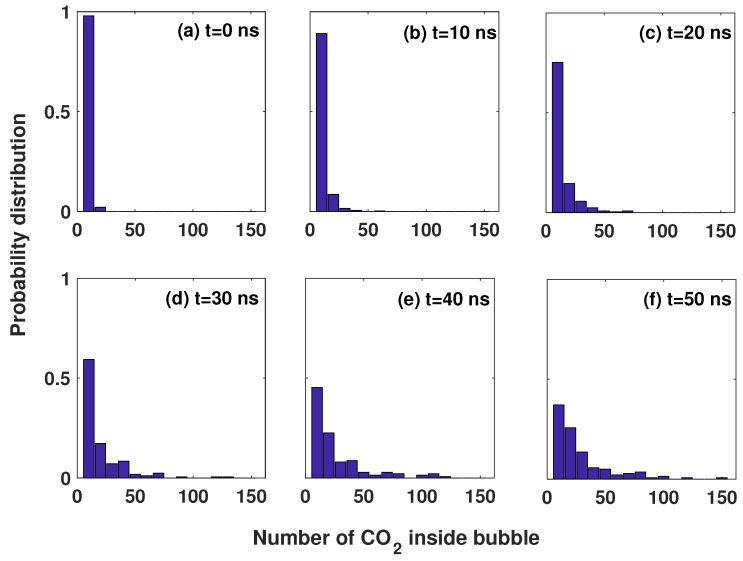
Probability distribution of number of CO${}_{2}$ molecules inside bubbles with a start-up pressure of 30 MPa.

Figure 6 shows the probability distribution of CO${}_{2}$ molecules in the simulation box at various times. In the pre-nucleation process (t=0,10,20 ns), most bubbles were small, with no more than 50 molecules. In the nucleation process, the bubbles became large by overcoming the nucleation barrier. Some bubbles were large, with more than 100 molecules; however, most bubbles were still small, with less than 50 molecules. By observing snapshots, we find that the small bubbles were in the region close to polymer chain, corresponding to the peak at ≈5 ${\mathrm{nm}}^{3}$ in Figure 5.

**Figure 7:**
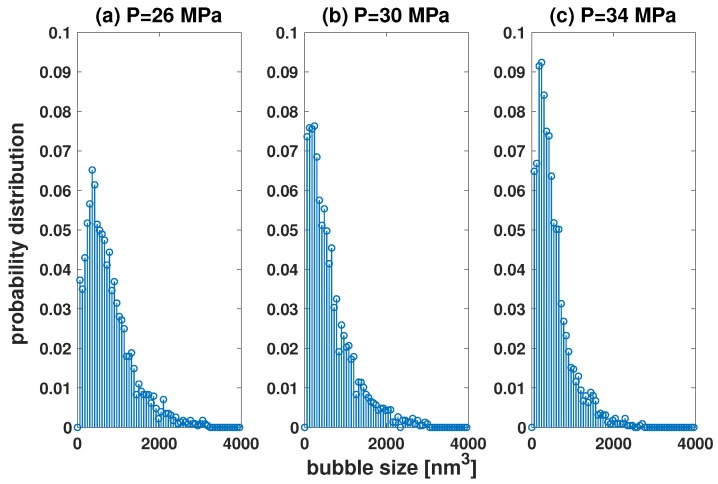
Probability distribution of bubble size with a startup pressure of (**a**) 26 MPa, (**b**) 30 MPa, and (**c**) 34 MPa.

**Figure 8:**
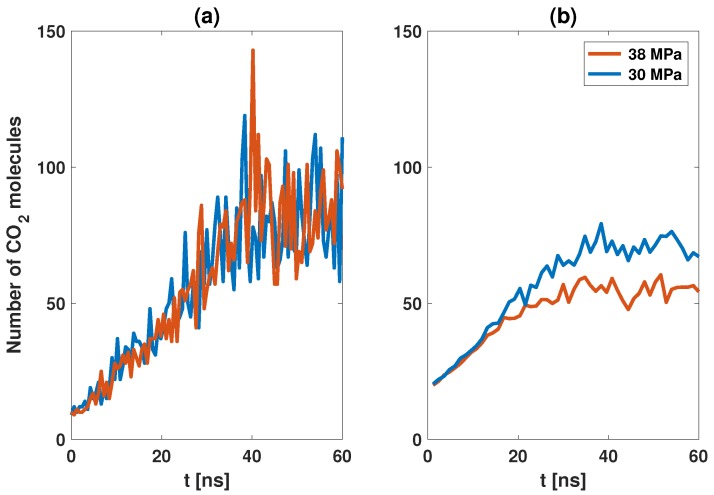
(**a**) Number of CO${}_{2}$ molecules in the biggest bubble. (**b**) Number of CO${}_{2}$ molecules exchanged between the bubble and the surroundings. We collected all data over a time interval of 1.2 ns.

Bubble size has a more uniform distribution with a higher start-up pressure [25] (see Figure 7). The reason for this was believed to be due to a stronger driving force at high pressure; which may explain why the bubble has a uniform size distribution in the nucleation stage. However, in the post-nucleation stage, the driving force of a well-developed bubble is almost the same for different start-up pressures. So, the real physical reason remains unclear. Here, we propose that the real reason is due to the existence of many small bubbles in the region close to the polymer chain. These small bubbles limit the dynamics of the CO${}_{2}$ molecules in a large bubble. Figure 8 shows the dynamic data in a bubble with maximal radius. The biggest bubble moves freely in the simulation box. Some molecules escape outside, and some molecules diffuse into the bubble. The number of molecules in the biggest bubble is almost the same at P=30 and 38 Mpa. However, the bubble at P=38 MPa is more stable than at P=30 MPa; see the data in panel (b). The number of CO${}_{2}$ molecules exchanged between bubble and surroundings at P=38 MPa is less than that at P=30 MPa.

Free CO${}_{2}$ molecules are the molecules that can move freely and are not a part of any bubble. At high pressures, there are many small bubbles in the region close to the polymer segments. The CO${}_{2}$ molecules in these small bubbles can not move freely, due to the entropy effect; that is to say, the existence of small bubbles limits the number of free CO${}_{2}$ molecules. Therefore, the big bubble becomes more stable at a higher pressure.

## 4. Conclusions

In this work, we explain why bubble size has a more uniform distribution with a higher start-up pressure. Free volume-rich polymer segments could adsorb many small size bubbles in the region close to polymer chain. The existence of these small bubbles limits the dynamics of the CO${}_{2}$ molecules in big bubbles, which are helpful for bubble stabilization. Moreover, the free volume of polymer segments decreases with an increase in start-up pressure; as a result, the size of the large bubbles becomes more uniform with a high start-up pressure.

## Figures and Tables

**Table 1:** Parameters for the species studied in this work.

Parameters	CO${}_{2}$	Bead A of PMMA ${}^{\left[1\right]}$	Bead B of PMMA ${}^{\left[1\right]}$
σ [nm]	0.3644	0.5829	0.5349
ϵ [kT]	231.7000	33.2746	76.7178

${}^{\left[1\right]}$ The bond lengths (AA and BB), bending angles (AAB and AAA) and torsional angles (AAAA, AAAB, and BAAB) are described by a group of probability distribution data; see [19] for details.

## References

[1] Xu X. (2010). Density-Functional Theory for Thermodynamic Properties of Polymers with Complex Chain Architecture. Ph.D. Thesis.

[2] Jacobs L., Kemmere M., Keurentjes J. (2008). Sustainable polymer foaming using high pressure carbon dioxide: A review on fundamentals, processes and applications. Green Chem..

[3] Kong W., Bao J., Wang J., Hu G., Zhao L. (2016). Preparation of open-cell polymer foams by CO_2_ assisted foaming of polymer blends. Polymer.

[4] Bienvenu Y. (2014). Application and future of solid foams. C. R. Phys..

[5] Lee S., Park C., Ramesh N. (2007). Polymeric Foams: Science and Technology.

[6] Xu X., Ting C., Kusaka I., Wang Z.-G. (2014). Nucleation in polymers and soft matter. Annu. Rev. Phys. Chem..

[7] Taki K. (2008). Experimental and numerical studies on the effects of pressure release rate on number density of bubbles and bubble growth in a polymeric foaming process. Chem. Eng. Sci..

[8] Leung S., Park C., Xu D., Li H., Robert G. (2006). Computer simulation of bubble-growth phenomena in foaming. Ind. Eng. Chem. Res..

[9] Li Y., Yao Z., Chen Z., Cao K., Qiu S., Zhu F., Zeng C., Huang Z. (2011). Numerical simulation of polypropylene foaming process assisted by carbon dioxide: Bubble growth dynamics and stability. Chem. Eng. Sci..

[10] Wang L., Zhou H., Wang X., Mi J. (2016). Evaluation of nanoparticle effect on bubble nucleation in polymer foaming. J. Phys. Chem. C.

[11] Xu X., Cristancho D.E., Costeux S., Wang Z.-G. (2012). Density-functional theory for polymer-carbon dioxide mixtures: A perturbed-chain SAFT approach. J. Chem. Phys..

[12] Zhang P., Wang X.-J., Yang Y., Zhou N.-Q. (2010). Bubble growth in the microcellular foaming of CO_2_/polyproplylene solutions. J. Appl. Polym. Sci..

[13] Yang W.J., Yeh H.C. (1966). Theoretical study of bubble dynamics in purely viscous fluids. AIChE J..

[14] Shima A., Tsujino T. (1976). The behaviour of bubbles in polymer solutions. Chem. Eng. Sci..

[15] Han C.D., Yoo H.J. (1981). Studies on structural foam processing. IV. Bubble growth during mold filling. Polym. Eng. Sci..

[16] Sahu S., Gokhale A., Mehra A. (2018). Effect of foaming temperature on bubble size distribution of liquid aluminium foam: Modeling and experimental studies. Trans. Indian Inst. Met..

[17] Mantilla I., Cristacho D., Ejaz S., Hall K., Atilhan M., Iglesias-Silva G. (2010). *P*-*ρ*-*T* data for carbon dioxide from (310 to 450) K up to 160 MPa. J. Chem. Eng. Data.

[18] Shin H.Y., Wu J.Z. (2010). Equation of state for the phase behavior of carbon dioxide polymer systems. Ind. Eng. Chem. Res..

[19] Chen C., Depa P., Maranas J., Sakai V. (2008). Comparison of explicit atom, united atom, and coarse-grained simulations of poly(methyl methacrylate). J. Chem. Phys..

[20] Depa P., Chen C., Maranas J. (2011). Why are coarse-grained force field too fast? A look at dynamics of four coarse-grained polymers. J. Chem. Phys..

[21] Van der Waals J.D. (1873). On the Continuity of Gaseous and Liquid States. Ph.D. Thesis.

[22] DeFelice J., Lipson J. (2014). Polymer miscibility in supercritical carbon dioxide: Free volume as a driving force. Macromolecules.

[23] Utraki L., Simha R. (2001). Free volume and viscosity of polymer-compressed gas mixtures during extrusion foaming. J. Polym. Sci. B Polym. Phys..

[B24-polymers-11-00648] 24.The probability shown in Figures 5–7 is the occurrence ratio of bubble over total number of bubble.

[25] Khan I., Adrian D., Costeux S. (2015). A model to predict the cell density and cell size distribution in nano-cellular foams. Chem. Eng. Sci..

